# CAMERA2 – combination antibiotic therapy for methicillin-resistant *Staphylococcus aureus* infection: study protocol for a randomised controlled trial

**DOI:** 10.1186/s13063-016-1295-3

**Published:** 2016-03-31

**Authors:** Steven Y. C. Tong, Jane Nelson, David L. Paterson, Vance G. Fowler, Benjamin P. Howden, Allen C. Cheng, Mark Chatfield, Jeffrey Lipman, Sebastian Van Hal, Matthew O’Sullivan, James O. Robinson, Dafna Yahav, David Lye, Joshua S. Davis, for the CAMERA2 study group and the Australasian Society for Infectious Diseases Clinical Research Network

**Affiliations:** Menzies School of Health Research, Charles Darwin University, Darwin, NT Australia; Royal Darwin Hospital, Darwin, NT Australia; University of Queensland, Centre for Clinical Research, Herston, QLD Australia; Division of Infectious Diseases, Department of Medicine, Duke University Medical Center, Durham, NC USA; Duke Clinical Research Institute, Duke University Medical Center, Durham, NC USA; Microbiological Diagnostic Unit Public Health Laboratory, The University of Melbourne, at The Doherty Institute, Melbourne, VIC Australia; Infection Prevention and Healthcare Epidemiology Unit, Alfred Health, Melbourne, VIC Australia; Department of Epidemiology and Preventive Medicine, Monash University, Melbourne, VIC Australia; Burns, Trauma Critical Care Research Centre, The University of Queensland, Brisbane, QLD Australia; Faculty of Health, Queensland University of Technology, Brisbane, QLD Australia; Department of Microbiology and Infectious Disease Royal Prince Alfred Hospital, Sydney, NSW Australia; Centre for Infectious Diseases and Microbiology, Westmead Hospital, Sydney, NSW Australia; Marie Bashir Institute for Infectious Diseases and Biosecurity, University of Sydney, Sydney, NSW Australia; Department of Microbiology and Infectious Diseases, Pathwest Laboratory Medicine WA, Royal Perth Hospital and Fiona Stanley Hospital, Perth, WA Australia; Australian Collaborating Centre for Enterococcus and Staphylococcus Species (ACCESS) Typing and Research, School of Veterinary and Life Sciences, Murdoch University, Perth, WA Australia; Faculty of Medicine, Tel Aviv University, Ramat Aviv, Israel; Rabin Medical Center, Petah Tikvah, Israel; Institute of Infectious Diseases and Epidemiology, Tan Tock Seng Hospital, Singapore, Singapore; Yong Loo Lin School of Medicine, National University of Singapore, Singapore, Singapore; John Hunter Hospital, Newcastle, NSW Australia

**Keywords:** Staphylococcus aureus, Methicillin-resistant, MRSA, Vancomycin, Combination, Randomised controlled trial, Daptomycin, Flucloxacillin, Cloxacillin, Cefazolin, Nafcillin

## Abstract

**Background:**

Methicillin-resistant *Staphylococcus aureus* (MRSA) bacteraemia is a serious infection resulting in 20–50 % 90-day mortality. The limitations of vancomycin, the current standard therapy for MRSA, make treatment difficult. The only other approved drug for treatment of MRSA bacteraemia, daptomycin, has not been shown to be superior to vancomycin. Surprisingly, there has been consistent in-vitro and in-vivo laboratory data demonstrating synergy between vancomycin or daptomycin and an anti-staphylococcal β-lactam antibiotic. There is also growing clinical data to support such combinations, including a recent pilot randomised controlled trial (RCT) that demonstrated a trend towards a reduction in the duration of bacteraemia in patients treated with vancomycin plus flucloxacillin compared to vancomycin alone. Our aim is to determine whether the addition of an anti-staphylococcal penicillin to standard therapy results in improved clinical outcomes in MRSA bacteraemia.

**Methods/Design:**

We will perform an open-label, parallel-group, randomised (1:1) controlled trial at 29 sites in Australia, New Zealand, Singapore, and Israel. Adults (aged 18 years or older) with MRSA grown from at least one blood culture and able to be randomised within 72 hours of the index blood culture collection will be eligible for inclusion. Participants will be randomised to vancomycin or daptomycin (standard therapy) given intravenously or to standard therapy plus 7 days of an anti-staphylococcal β-lactam (flucloxacillin, cloxacillin, or cefazolin). The primary endpoint will be a composite outcome at 90 days of (1) all-cause mortality, (2) persistent bacteraemia at day 5 or beyond, (3) microbiological relapse, or (4) microbiological treatment failure. The recruitment target of 440 patients is based on an expected failure rate for the primary outcome of 30 % in the control arm and the ability to detect a clinically meaningful absolute decrease of 12.5 %, with a two-sided alpha of 0.05, a power of 80 %, and assuming 10 % of patients will not be evaluable for the primary endpoint.

**Discussion:**

Key potential advantages of adding anti-staphylococcal β-lactams to standard therapy for MRSA bacteraemia include their safety profile, low cost, and wide availability.

**Trial registration:**

ClinicalTrials.gov Identifier: NCT02365493. Registered 24 February 2015.

## Background

*Staphylococcus aureus* is one of the most important human bacterial pathogens, and causes a broad range of infections, ranging from superficial skin infections, deep skin and tissue abscesses and bone infections, to invasive bloodstream infections [1]. Methicillin-resistant *S. aureus* (MRSA) is resistant to the mainstay of *S. aureus* therapy, the anti-staphylococcal penicillins (such as flucloxacillin) and is hence more difficult to treat. Therapies for MRSA are either less effective (e.g. vancomycin) or much more expensive (e.g. daptomycin) than the anti-staphylococcal penicillins are for methicillin-susceptible *S. aureus*. Alternative therapies, including novel combinations of existing agents are, therefore, urgently required, particularly to treat invasive MRSA infections, each episode of which results in a mortality of 20–50 % [2].

### Invasive MRSA infection causes a substantial burden of disease

The Australian and New Zealand Co-operative Outcomes of Staphylococcal Sepsis (ANZCOSS) study included data from 33 hospitals and found that of 10,085 *Staphylococcus aureus* bacteraemia (SAB) cases in 6 years (2007–2012), 2881 (22 %) were MRSA with an average of 480 MRSA bacteraemia (MRSA-B) cases per year [2]. Although data are lacking, the disease burden is likely to be even higher in large population centres in Asia as indicated by high case numbers in hospitals in Singapore. Although hospital-acquired MRSA infections have decreased in the US, UK and Australia with improved infection control practices, community-associated strains of MRSA have emerged in the past 10–15 years and the majority of invasive MRSA infections are now community-onset rather than nosocomial [3]. This is reflected in ANZCOSS data, where community-onset cases of MRSA-B (index blood culture taken earlier than 72 hours following admission) increased from 51 % in 2007 to 69 % in 2012. Attempts to prevent MRSA infections outside the hospital system are unlikely to be effective, and further reductions in the incidence of MRSA infections are not expected.

### MRSA-B is associated with poor outcomes

Bloodstream infections with MRSA have a higher mortality than those caused by methicillin-sensitive *Staphylococcus aureus* (MSSA) [4]. The ANZCOSS dataset demonstrates that 30-day mortality is higher at 24 % for MRSA compared to 17 % for MSSA (*P* <0.001). In a Thai hospital, mortality rates were 67 % and 46 % for MRSA and MSSA respectively [5]. This high mortality, not only in Australia, Singapore, New Zealand and Israel, but also in resource-limited settings where SAB is common and infection control practices are suboptimal, is a key reason for the currently described randomised controlled trial (RCT).

### Current therapies for MRSA-B are limited and associated with poor outcomes

A significant factor contributing to poorer outcomes with MRSA-B compared to methicillin-susceptible *S. aureus* bacteraemia (MSSA-B) is the limitations of vancomycin (the current standard antibiotic therapy for invasive MRSA infections). Compared with anti-staphylococcal β-lactams such as oxacillin and its derivatives (flucloxacillin, cloxacillin, and nafcillin), vancomycin demonstrates slower bacterial killing [6], poorer tissue penetration [7], slower clearance of bacteraemia [8, 9] and higher mortality [10, 11]. For MSSA bacteraemia in ANZCOSS, 30-day mortality was 21 % (133/638) and 12 % (937/6950) for those treated with vancomycin or β-lactams respectively. Furthermore, treatment with vancomycin compared to β-lactams was a risk factor for 30-day mortality among all participants with SAB, independent of MRSA versus MSSA status (*P* <0.001) [2]. In addition, strains of MRSA with decreased susceptibility to vancomycin (heterogeneous vancomycin intermediate resistance *S. aureus* (hVISA)) are beginning to emerge worldwide [12]. In recent years, several alternative agents to vancomycin have become available for the treatment of MRSA-B, including linezolid, daptomycin and ceftaroline. Each of these has been found to be non-inferior to vancomycin for MRSA infections, but none have been shown to be superior [13] and all are associated with a high cost and a substantial risk of adverse effects [14]. Thus vancomycin continues to be recommended as the first-line agent for severe MRSA infections by both the Infectious Diseases Society of America [15] and the Australian Therapeutic Guidelines: Antibiotic [16]. Ceftaroline has only recently become available for MRSA pneumonia and skin infections, but no trials have yet been completed comparing it with vancomycin for MRSA-B. However, even if ceftaroline were to eventually prove more effective, its cost far exceeds that of vancomycin (estimated drug cost for a 4-week course of ceftaroline = A$8680, compared with vancomycin = A$260). Ceftaroline resistance is an additional concern with a recent Australian study finding overall resistance rates of 17 % amongst MRSA and 41 % in sequence type 239 MRSA [17].

An alternative strategy to improve outcomes from MRSA-B is to combine vancomycin with a second agent, aiming for synergistic bacterial killing [18, 19]. Neither linezolid nor daptomycin demonstrate synergy with vancomycin against MRSA [19]. However, β-lactam antibiotics, to which MRSA is inherently resistant, demonstrate an unexpected but consistent synergy with vancomycin and daptomycin respectively against MRSA. Given that β-lactams are cheap (e.g. 7 days of flucloxacillin costs $A47), safe and widely available, they are an attractive alternative to more expensive drugs as second agents to combine with vancomycin.

### Previous studies of β-lactam combination therapy

Due to poor outcomes with vancomycin monotherapy, and the emerging problem of decreased vancomycin susceptibility in MRSA [20], multiple research teams have investigated the combination of vancomycin or daptomycin with various β-lactam antibiotics (reviewed in detail in Davis et al. [21]).

At least 16 in-vitro studies have explored synergy between vancomycin and β-lactams against MRSA isolates [22–37], all but one of which found evidence of synergy in some or all of the tested strains. These studies varied in their methodology (checkerboard synergy testing or time-kill curves), types of strains tested (MRSA versus hVISA versus vancomycin intermediate *Staphylococcus aureus* (VISA)) and the β-lactams used, but a consistent finding across nearly all the studies was synergistic bacterial killing in *most* but not *all* strains tested. There was a general tendency across these studies (and *within* some studies [24, 34]) to an increasing degree of synergy with increasing vancomycin minimum inhibitory concentrations (MICs). Synergy has been reported with all β-lactams tested (including cefazolin), but the largest effect has been observed with oxacillin and nafcillin. Flucloxacillin is also considered in the same antibiotic class of ‘anti-staphylococcal semi-synthetic penicillins’.

The few studies that have assessed combinations of vancomycin with β-lactams in animal models have all found evidence of synergy [24, 29, 33]. Climo found faster sterilisation of infection with vancomycin plus nafcillin in MRSA rabbit endocarditis and renal abscess models [24]. Ribes tested various combinations of linezolid, vancomycin and imipenem in a murine peritonitis VISA model using time-kill curves, and found faster bacterial killing with vancomycin plus imipenem compared with vancomycin alone, in both strains tested [29]. Finally, Fernandez investigated the anti-MRSA cephalosporin ceftobiprole against an MRSA and a VISA strain in a rat endocarditis model. They found good activity of ceftobiprole against both strains in terms of sterilising vegetations and preventing mortality; the combination of vancomycin plus ceftobiprole led to faster killing on time-kill curves, but similar rates of mortality and of sterilisation of vegetations compared with ceftobiprole alone [33].

There are few published data on β-lactam-based combination therapy for MRSA in humans. In a single-centre retrospective cohort study, Dilworth and colleagues described the outcomes of 50 participants with MRSA-B who received combination therapy with vancomycin and at least 24 hours of β-lactam (at their clinicians’ discretion), and compared them with 30 participants treated at the same hospital, during the same time period with vancomycin alone [35]. They found a higher rate of microbiological eradication in the combination therapy group (96 % versus 80 %, *P* = 0.02), which persisted on a multivariate model attempting to control for potential confounders (adjusted odds ratio for achieving microbiological eradication in the combination group = 11.24, *P* = 0.01).

In the only prospective clinical trial to date (CAMERA1), Davis et al. [38] randomised 60 patients with MRSA-B to standard therapy with vancomycin alone, or to combination therapy with vancomycin and flucloxacillin. The study was conducted in seven centres in Australia and was open-labelled in design. Patients receiving combination therapy cleared bacteraemia at a mean of 2 days compared to 3 days with standard therapy (*P* = 0.06).

At least 10 in-vitro studies have examined the combination of daptomycin with various β-lactams against MRSA and VISA strains [39–49]. The findings of these studies are remarkably similar to the vancomycin/β-lactam synergy papers cited above: synergy for most but not all strains tested, and an increasing degree of synergy with increasing MICs to both vancomycin and daptomycin. No studies have found evidence of antagonism with this combination. A recently published animal study mirrored the findings of the in-vitro studies. Garrigos used a rat tissue cage model of MRSA infection to study the combination of daptomycin with cloxacillin, and found superior cure rates with the combination than with daptomycin alone [50].

As for the vancomycin/β-lactam combination, there are no clinical trials of daptomycin with β-lactams either published or in trials registries. However, limited observational data suggest this combination may be effective, particularly MRSA with poor response to daptomycin. In a case series of seven participants with persistent MRSA-B for more than 1 week despite high-dose daptomycin, all had their bacteraemia cleared within 48 hours once naficillin or oxacillin was added to their therapy [51]. In a second case series of 22 participants with persistent MRSA-B despite daptomycin for a median of 10 days, the addition of ceftaroline led to clearance of bacteraemia in all cases, in a median of 2 days [52].

A key question that emerges from these data is: what is the mechanism of the observed synergy? The mechanisms have not been entirely elucidated, but are becoming clearer over time. Increasing vancomycin resistance in *S. aureus* is paradoxically associated with decreasing MICs to oxacillin, and this so-called ‘see-saw effect’ [36, 53] is at least in part due to deletion of the *MecA* gene in some strains of VISA and vancomycin-resistant *Staphylococcus aureus* (VRSA) [54, 55], and possibly to other structural changes in penicillin-binding proteins and cell wall thickness. β-Lactams have been shown to enhance binding of daptomycin to the bacterial cell wall [49]. Finally, Sakoulas et al. recently reported novel data derived from an ex-vivo study of human blood which adds another potential advantage for the use of β-lactams for MRSA – they lead to increased activity of innate host defence peptides such as cathelicidin LL-37 [56], which in turn allow more efficient bacterial killing.

Thus there is considerable in-vitro and in-vivo and growing clinical evidence that the combination of vancomycin or daptomycin with a β-lactam may be more effective than vancomycin or daptomycin alone for improving outcomes of this common and devastating infection.

### Hypothesis

We hypothesise that the addition of β-lactams to standard therapy in adults with MRSA-B will lead to synergistic bacterial killing and hence faster clearance of bacteria from the bloodstream and other infected foci, thereby reducing the risk of disseminated infection and death.

### Primary objective

To determine whether 7 days of intravenous β-lactam given intravenously (IV) in combination with standard therapy will lead to better 90-day complication-free survival, compared to standard therapy alone in adult participants with MRSA bloodstream infection.

## Methods/Design

### Overview of trial design

CAMERA2 is an investigator-initiated, multi-centre, parallel-group, open-label, RCT powered for superiority, which compares combination antibiotic therapy with standard antibiotic therapy in adults with MRSA-B. Ethical approval has been obtained from all relevant Human Research Ethics Committees (HRECs) and Institutional Review Boards (see Appendix for details).

### Study setting

We are planning to recruit from 23 Australian, 1 New Zealand, 3 Singaporean and 2 Israeli acute care hospitals. Other sites may be added during the course of the study. Sites have been selected on the basis of (1) their incidence of MRSA-B (at least 10 cases per year and ideally 20), (2) the availability of an experienced and committed site principal investigator (PI), and (3) the availability of a suitably qualified research nurse *or* senior registrar to assist with study-related activities.

### Eligibility criteria

#### Participant inclusion criteria

1. Age 18 years or older
2. At least one set of blood cultures positive for MRSA
3. Able to be randomised within 72 hours of blood culture being collected
4. Likely to remain as an inpatient for 7 days following randomisation

#### Participant exclusion criteria

1. Previous type 1 hypersensitivity reaction to β-lactams
2. Mixed blood culture with more than one pathogen (excluding contaminants – i.e. a mixed growth of MRSA and coagulase-negative *Staphylococcus* (CNS) is eligible, as long as the CNS is clinically judged to be a likely contaminant)
3. Previous participation in the trial
4. Known pregnancy
5. Current β-lactam antibiotic therapy which cannot be ceased or substituted
6. Patient’s primary clinician unwilling to enrol patient
7. Moribund (expected to die in next 48 hours with or without treatment)
8. Treatment limitations that preclude the use of antibiotics. Participants who are ‘not for resuscitation’ or ‘not for ICU admission’ may still be enrolled if they are for active management of infection including the use of all necessary antibiotics and intravenously administered fluids.

### Interventions

#### Standard care arm

Either vancomycin given IV dosed in accordance with the Australian Therapeutic Guidelines: Antibiotic version 15, 2014 [16] (15–20 mg/kg 12-hourly (q12h), preceded by a loading dose of 20–35 mg/kg if considered appropriate by the treating clinician) or the Infectious Diseases Society of America (IDSA) guidelines [57] with subsequent adjustment to maintain trough levels at 15–20 mg/dL *or* daptomycin 6–10 mg/kg per day by intravenous infusion (IVI) (both drugs will be adjusted for renal function, see Tables 1, 2, 3, 4 and 5). Dosing of vancomycin may follow local guidelines if broadly in line with the Australian Therapeutic Guidelines: Antibiotic and the IDSA guidelines. The choice of vancomycin or daptomycin will be at the clinician’s discretion. The non-antibiotic management and duration of the intravenously administered vancomycin or daptomycin will be at the clinicians’ discretion, but will be in line with Australian Therapeutic Guidelines and IDSA guidelines [15]. These recommend from 14 to 42 days of intravenous treatment, depending on factors such as the result of a blood culture at 2 to 4 days after index blood culture, result of echocardiogram, and the presence and removal of a focus of infection.

**Table 1:** Adjustment of starting maintenance vancomycin doses according to renal function (for a 70-kg adult)

Creatinine clearance (mL/min)	Starting maintenance dosage	Timing of trough (pre-dose) plasma concentration measurement
More than 90	1.5 g 12-hourly	Before the fourth dose
60 to 90	1 g 12-hourly	Before the fourth dose
20 to less than 60	1 g 24-hourly	Before the third dose
Less than 20	1 g 48-hourly	48 hours after the first dose
On haemodialysis [58]	25 mg/kg	Immediately prior to next haemodialysis session

**Table 2:** Adjustment of ongoing vancomycin doses for those on haemodialysis

Vancomycin level (mg/L)	Next vancomycin dose (mg)
<5	2000
5–15	1500
15–20	1000
20–25	500
>25	0

**Table 3:** Adjustment of (flu)cloxacillin doses according to renal function

GFR	Flucloxacillin dose	Cloxacillin dose
>50 ml/min	2 g q6h IVI	2 g q6h IVI
11–50 ml/min	2 g q6h IVI	2 g q6h IVI
≤10 but not on haemodialysis	1 g q8h IVI	2 g q6h IVI
On continuous renal replacement therapy	2 g q6h IVI	2 g q6h IVI
On haemodialysis	Not for flucloxacillin (cefazolin 2 g 3x/week)	Not for cloxacillin (cefazolin 2 g 3x/week)

**Table 4:** Adjustment of cefazolin doses according to renal function

GFR	Cefazolin dose
>40 ml/min	2 g q8h IVI
21–40 ml/min	1 g q8h IVI
≤20 but not on haemodialysis	1 g q12h IVI
On continuous renal replacement therapy	2 g q12h IVI
On haemodialysis	2 g 3x/week post dialysis

**Table 5:** Adjustment of daptomycin doses according to renal function

GFR	Daptomycin dose
>50 ml/min	6–10 mg/kg IVI q24h
11–50 ml/min	6–8 mg/kg q24h IVI
≤10 but not on haemodialysis	8 mg/kg q48h IVI
On continuous renal replacement therapy	8 mg/kg q48h IVI
On haemodialysis	8 mg/kg q48h IVI, dose after dialysis

GFR *glomerular filtration rate, IVI intravenous infusion,* q6/8/12h *6/8/12-hourly*

#### Combination therapy arm

In addition to standard treatment, a β-lactam given IV will be added for the first 7 calendar days following randomisation (day 1 being the day of randomisation – hence patients will receive 6–7 days of β-lactam). This β-lactam will be flucloxacillin 2 g q6h IVI in Australia and New Zealand, and cloxacillin 2 g q6h IVI in Singapore and Israel (where flucloxacillin is not generally available). For those with a history of minor allergy to any penicillin (rash or unclear history, but not anaphylaxis or angioedema), it will be cefazolin 2 g q8h IVI. For haemodialysis patients, it will be cefazolin 2 g three times per week post dialysis.

### Criteria for discontinuing or modifying allocated interventions

#### Adjusting for renal function

The starting maintenance vancomycin dose will be guided by Table 1.

For those on haemodialysis, blood is to be taken at the commencement of each dialysis session and sent for an urgent vancomycin level. The dose as per the nomogram (Table 2) is then administered and timed for the vancomycin infusion to be completed simultaneously with the completion of dialysis.

Other antibiotic doses will be adjusted for renal function as per Table 3 ((flu)cloxacillin), Table 4 (cefazolin), and Table 5 (daptomycin).

#### Change of ‘backbone drug’ (vancomycin or daptomycin) after randomisation

Whilst unnecessary changes will be discouraged, it will be left to the treating clinician’s discretion to switch these drugs if needed. The most likely situation where a switch might occur is if a patient is commenced on vancomycin, but the vancomycin MIC of the MRSA isolate is later determined to be ≥1.5 μg/ml. If a patient develops a suspected adverse drug reaction to daptomycin (e.g. raised serum creatinine kinase (CK)) or vancomycin (e.g. rash), then clinicians may also choose to switch.

If a patient’s backbone drug is switched, they will still be analysed in the group to which they were randomised (standard or combination). In the subgroup analysis (vancomycin vs daptomycin), they will be counted as the drug which they received the majority of doses of in the first 7 days post randomisation. For example, if a patient switches from vancomycin to daptomycin on day 3, they will be counted in the daptomycin group.

Switching to a backbone drug other than vancomycin or daptomycin will be discouraged. If a participant is switched to another non-β-lactam backbone drug (e.g. linezolid, cotrimoxazole, clindamycin, tigecycline, quinupristin-dalfopristin) this will be a protocol deviation, but they will continue on the study and will still be analysed in the group to which they were randomised (standard or combination). Switching the backbone drug to ceftaroline (a β-lactam with anti-MRSA activity) at any time in the first 90 days will be a protocol violation, but the participant will remain in the study and be analysed in the group to which they were randomised, but will be excluded from the per-protocol analysis (in accordance with criteria in section 2.10)

#### β-lactam use after randomisation

Standard therapy group: the use of all β-lactams will be prohibited in participants allocated to the standard therapy group for the first 14 days after randomisation, and will be discouraged for the entire duration of intravenously administered vancomycin/daptomycin. If a patient develops an indication for broadening of antibiotic therapy, the site PI should recommend a non-β-lactam agent (e.g. clindamycin, quinolones). If a patient allocated to the standard therapy group receives a β-lactam within the first 14 days post randomisation in spite of this, this will be recorded as a protocol violation, but will remain in the study.

Combination therapy group: the β-lactam may be switched (within the limits of flucloxacillin, cloxacillin and cefazolin) by the patient’s clinician if there is a serious clinical need to do so (e.g. suspected allergy or toxicity). The β-lactam must be ceased at the end of day 7.

### Strategies to improve adherence to protocol

#### Training of site principal investigators (PIs)

All site PIs will receive training with regards to the study protocol and their reporting requirements by the project manager, a study chief investigator (CI) or delegate, prior to the site being opened for recruitment. All site PIs will complete a computer-based training course in Good Clinical Practice (GCP). The project manager or delegate will have regular phone contact with all enrolling site investigators.

#### Checking of medication charts

The medication chart (be it paper or electronic) will be checked regularly by the site PI or their delegate (registrar or research nurse) for the first 14 days whilst an inpatient to ensure adherence to the study protocol.

### Outcomes

#### Primary outcome – complication-free 90-day survival

The primary outcome is a composite outcome measure with four components, to be assessed 90 days after randomisation (randomisation = day 1). These are any of:

1. All-cause mortality
2. Persistent bacteraemia at day 5 or beyond
3. Microbiological relapse – positive blood culture for MRSA at least 72 hours after a preceding negative culture
4. Microbiological treatment failure. Positive sterile site culture for MRSA at least 14 days after randomisation. This includes pus from deep tissue or organ abscesses, synovial fluid, blood or other normally sterile sites. It does not include urine, sputum or superficial swabs

#### Secondary outcomes

All outcomes below refer to the time period from randomisation to day 90:

1. All-cause mortality at days 14, 42 and 90 days
2. Persistent bacteraemia at day 2
3. Persistent bacteraemia at day 5 or beyond
4. Acute kidney injury defined as at least stage 1 modified RIFLE criteria (1.5-fold increase in the serum creatinine, or glomerular filtration rate (GFR) decrease by 25 %) at any time within the first 7 days, *or* new need for renal replacement therapy at any time from days 1 to 90. This endpoint does not apply to participants who were already on haemodialysis at randomisation
5. Microbiological relapse – positive blood culture for MRSA at least 72 hours after a preceding negative culture
6. Microbiological treatment failure. Positive sterile site culture for MRSA at least 14 days after randomisation
7. Duration of intravenously administered antibiotic treatment
8. Direct healthcare costs

### Rationale for these outcome measures

#### Primary outcome measure

Whilst the key outcome of interest is all-cause mortality, a study powered to detect a clinically meaningful 5 % absolute mortality reduction would require over 2000 participants, which is beyond the capacity of this study. Hence a composite outcome measure incorporating mortality and microbiological measures of treatment failure has been chosen. Clinical assessments of treatment failure have been avoided due to their subjective nature. Since there exists no generally agreed upon outcome measure for SAB trials, we generated the primary outcome measure according to the following principles – we chose an outcome that was: patient-centred and clinically meaningful; as objective as possible; simple to measure with as small a departure as possible from usual clinical processes; consensus from a group of experts (the CIs) following repeated cycles of assessment, discussion and reassessment; consistent with outcomes used in contemporary RCTs (e.g. the ARREST trial of adjunctive rifampicin for SAB [58]).

The 90-day post-randomisation time point was chosen because the majority of participants will have completed their initial course of orally- and intravenously-administered antibiotic treatment by this time; using 28-day mortality may miss an important proportion of infection-related mortality and hence later time points are increasingly used [58].

#### Secondary outcome measures

Each component of the composite primary outcome measure has been included as a secondary outcome measure. In addition, we have included acute kidney injury (defined according to the validated RIFLE criteria [59]). This is because several small studies have raised the possibility of vancomycin plus β-lactam combinations being nephrotoxic [60, 61], although both the cited studies involved piperacillin-tazobactam as the β-lactam.

### Endpoint assessment

The composite primary endpoint will be assessed by a blinded Endpoint Adjudication Committee. This committee will consist of three infectious diseases physicians (IDPs), to be appointed by the Trial Management Committee. This committee will be provided with an extract of study data that does not contain patient identifiers, and does not contain any mention of treatment allocation or any detail about antibiotic treatment, but does contain:

1. Demographic details (such as age and sex)
2. Comorbidities
3. Clinical details (including focus of infection, Sepsis-related Organ Failure Assessment (SOFA) scores, and echocardiography results)
4. Date and result of all blood cultures taken from the index blood culture through to study day 90
5. Date and result of all other available clinical cultures taken from days 1–90 (e.g. cultures of aspirated pleural fluid or pus)
6. Vital status at day 90 and date of death if applicable

The members of the committee may request more information if needed, but this will only be provided if it is available and does not provide direct or indirect evidence of treatment allocation. Each of the three members of the committee will then independently determine if, in their view, the patient has met the primary endpoint. If there is a discrepancy between the three assessments, the majority will determine the endpoint.

### Participant timeline

**Fig. 1:**
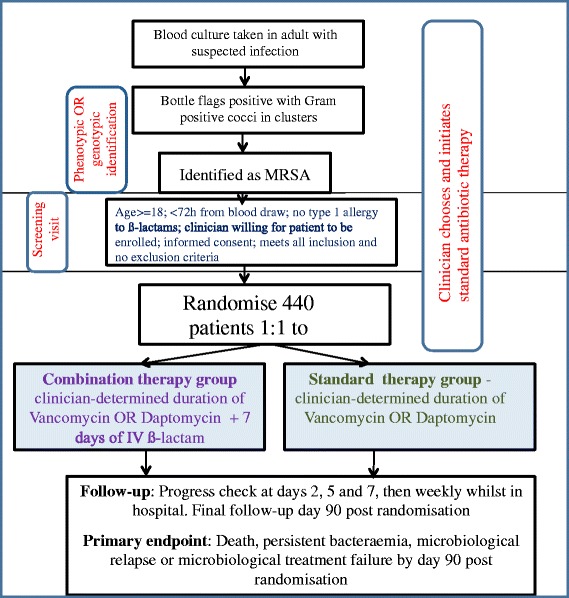
Trial flowchart

See Fig. 1 and Table 6 for a summary of participant procedures.

**Table 6:** Schedule of visits, data collection and follow-up

Visit day	Pre-screen	Day 1	Day 2	Day 3	Day 4	Day 5	Day 6	Day 7	Days 8–13	Day 14	Day 15–41	Day 42	Days 43–89	Day 90
Check eligibility	x													
Informed consent		x												
Demographic data		x												
Clinical details		x												
Randomise		x												
Ensure blood cultures are ordered by treating clinicians	x		x			x		(x)^a^	(x)^a^	(x)^a^	(x)^a^			
Ensure FBC, EUC, LFTs, CRP and vancomycin levels are ordered by treating clinicians			x			x		x	As clinically indicated					
Vancomycin *or* daptomycin doses		x	x	x	x	x	x	x	x	x	(x)^b^	(x)^b^	(x)^b^	
Combination therapy group: β-lactam doses		x	x	x	x	x	x	x						
Clinical progress assessment			x			x		x		x	Weekly whilst in hospital			x
Vital status (alive)		x		x		x		x		x		x		x
Additional data review									x	x	x	x		x

*CRP* C-reactive protein**,** *EUC* electrolytes urea and creatinine**,** *FBC* full blood count**,** *LFTs* liver function tests

^a^If blood cultures are still positive at day 5, they should be recollected on day 7 and then every 48 hours until negative.

If they are negative on day 5, they should be recollected only if there is any clinical suspicion of relapse (eg. recurrent fever)

^b^Minimum recommended duration of vancomycin or daptomycin is 14 days – clinicians may choose to give longer courses, typically up to 42 days

### Sample size

We have estimated that the failure rate for the primary outcome in the control group will be 30 % (as per data from the CAMERA1 study). We are aiming to detect a clinically meaningful absolute reduction in failure by 12.5 %. The absolute risk reduction we want to detect is based on what is considered clinically significant – which is a subjective quantity, based on expert opinion. When CIs of CAMERA2 were asked about this, the answers ranged from 10 to 15 %. Hence we have arbitrarily taken the midpoint of 12.5 %, resulting in a sample size required of 438 (including 11.1 % inflation for 10 % drop out). A trial of 394 participants with complete data for the primary outcome will provide 83 % power to detect a statistically significant difference at the two-sided 5 % level. We will, therefore, aim to randomise 440 participants to allow for approximately 10 % drop out and have at least 394 participants for analysis.

### Assignment of interventions

#### Allocation

Participants will be randomised in a 1:1 ratio to the standard or combination treatment arms, using a web-based interactive randomisation system, available 24 hours per day, 7 days per week (Spiral Software, Wellington, New Zealand). Randomisation will be stratified by site, and by receipt of haemodialysis, and will be in permuted blocks of variable block size.

#### Allocation concealment

The randomised sequence allocation will be stored on a secure server, and will not be available to any investigators or member of study staff.

#### Implementation

A commercial provider of randomisation services (Spiral Software, Wellington, New Zealand) will generate the allocation sequence and store it on their secure servers. Participants will be enrolled by site PIs or their delegates (research nurse or co-investigator (Co-I)). The person enrolling the patient will, following obtaining written informed consent, obtain the treatment allocation by logging onto the web-based database and will then assign the allocated treatment to the patient.

### Blinding

This will be an open-label study, but the Outcome Adjudication Committee assessing the primary outcomes will be blinded to treatment allocation.

### Data management

#### Source data

Source documents are those where data are first recorded, and from which participants’ case report form (CRF) data are obtained. These include but are not limited to hospital records both electronic and paper (which will include medical history, previous and current medications, any relevant radiography test, blood test results, haemodynamic parameters and medical correspondence) and paper or electronic clinic records (which will include vital status, recent medical history and relevant blood culture results). A further potential data source will be through telephone conversations with the study participant, person responsible or GP.

Storage and archiving of study documents (CRFs and consent forms) will be the responsibility of the site PI and these will remain at the site of recruitment. All study participants will be allocated a unique number at time of screening (screening number); this screening number will be added to all the CRFs for that participant. The participants will also have their hospital record number (HRN) recorded on the CRFs as this information will be required to ensure the correct medical record is accessed during medical record reviews.

#### Data recording and record keeping

Data for this study will be recorded via a secure, Electronic Data Capture (EDC) web-based system. It will be transcribed by the site PI or their delegate from the paper CRFs onto the EDC*.* Data will be stored in a re-identifiable manner in the database, using a unique screening number for each patient. The database will contain validation ranges for each variable to minimise the chance of data entry errors. An audit trail will maintain a record of initial entries and changes made; reasons for change; time and date of entry; and user name of person who made the change. Data queries will be raised by the project manager and study monitor, and missing data or suspected errors will be raised as data queries and resolved prior to database lock and analysis. The database will contain in-line capability so that these queries and answers are logged as part of the audit trail.

The Trial Steering Committee will be the custodians of the final trial dataset. No-one outside the Trial Steering Committee will be given access to the data without the permission of the Trial Steering Committee. No identifying data will be given to any third parties at any stage. Following study close out and locking of the database, it will be stored on the servers of the Menzies School of Health Research.

### Statistical methods

#### Statistical analysis plan

Data will be reported in accordance with the Consolidated Standards of Reporting Trials (CONSORT) guidelines for reporting of randomised trials. Proportions will be compared between treatment groups with Fisher’s exact or χ^2^ tests, and the absolute difference in proportions reported with corresponding 95 % confidence intervals. All-cause mortality will be presented in a Kaplan-Meier graph.

The *primary analysis* of both primary and secondary endpoints will be according to modified intention-to-treat principles (all participants with data available for the endpoint will be analysed according to the treatment allocation, regardless of what treatment they received).

A *secondary per-protocol analysis* of all endpoints will be conducted. The per-protocol population is defined as (1) for the combination group: received at least 75 % of β-lactam doses, (2) for the standard treatment group: received at least one defined daily dose of β-lactam, (3) has available day-90 data. For example, a patient who was allocated to flucloxacillin 2 g four times a day (QID) for 7 days (28 doses in total), must receive at least 21 doses during the first 7 days to be included in the per-protocol population. A patient on haemodialysis three times per week who is prescribed cefazolin 2 g post dialysis, must have received at least three doses in the first 7 days (i.e. have missed no doses if dialysed three times on days 1–7 or a maximum of one dose if dialysed four times on days 1–7). We will perform the following subgroup analyses:

1. *Standard treatment was daptomycin versus vancomycin*. This is because it is possible that daptomycin and vancomycin are not equivalent in terms of the primary outcome. Even though at least one previous RCT has directly compared them and found daptomycin to be non-inferior to vancomycin, there was a trend towards improved success with daptomycin for the MRSA subgroup [62]. Similarly, the synergistic effect of a β-lactam may differ depending on the backbone drug
2. *Vancomycin MIC of primary isolate***≥***1.5 μg/ml, or <1.5 μg/ml*. Synergy between β-lactams and vancomycin or daptomycin appears to be more pronounced in isolates with higher vancomycin MICs. Conversely, higher vancomycin MICs have been associated with worse outcomes, including higher mortality [63]. The difference between the combination therapy group and the standard therapy group is likely to be larger (in the direction of benefit) in those with a higher vancomycin MIC
3. *Participants receiving intermittent chronic haemodialysis compared with those who are not*. Haemodialysis participants may have worse outcomes from MRSA-B than those not on haemodialysis, and they will be receiving a different β-lactam regimen than others (cefazolin three times per week rather than (flu)cloxacillin four times daily). Hence the benefit of combination therapy may be smaller in those on haemodialysis
4. *Those who received more than 24 hours of β-lactam antibiotics within the 72 hours prior to randomisation compared with those who did not*. The effect of any intervention for MRSA-B is likely to be greatest within the first 24–48 hours after onset. The benefit of combination therapy is likely to be smaller in those who have received β-lactams prior to randomisation, because of a dilution of effect (the control group having received the intervention for a time)
5. *Uncomplicated versus complicated SAB* (uncomplicated SAB defined as per IDSA guidelines: exclusion of endocarditis; no implanted prostheses; follow-up blood cultures performed on specimens obtained 2–4 days after the initial set that do not grow MRSA; defervescence within 72 hours of initiating effective therapy; and no evidence of metastatic sites of infection) [15]. Complicated SAB participants have worse outcomes and longer durations of bacteraemia. The effect of combination therapy is likely to be larger in this group. Because we expect the combination therapy arm to result in a shorter duration of bacteraemia and thus fewer patients to have positive blood cultures at days 2–4, we will also use an a-priori definition of uncomplicated SAB that does not include the blood culture criteria at days 2–4
6. *Participants recruited in Australia/New Zealand versus Singapore versus Israel*. We expect that approximately 50 % of patients will be recruited from Singapore
7. *Those with baseline immunosuppression versus those without*. These are different patient groups with regards to underlying comorbidities and risk for severe sepsis
8. *Participants with endocarditis affecting the left side of the heart versus those without*. Those with endocarditis affecting the left side of the heart generally have a poorer prognosis than those without endocarditis
9. *Participants with community-associated MRSA versus healthcare-associated MRSA* (defined either genotypically or by non-multi (nmMRSA) versus multidrug-resistant (mMRSA) phenotype; nmMRSA defined as resistant to fewer than three classes of non-β-lactam antibiotics, and mMRSA as resistance to at least three classes of non-β-lactam antibiotics).

A simple *health economic analysis* will also be carried out, using the primary outcome measures for the trial to inform a modelling study. We will make use of cost and quality of life estimates from other studies/data sources.

### Interim analyses and stopping guidelines

The Data Safety and Monitoring Board (DSMB) will conduct an interim analysis after 220 patients have been randomised and followed for 90 days, *or* 2 years following the date of the first patient randomised, whichever comes first.

The interim analysis will review outcome data and answer the following questions:

1. Are there any significant safety issues that may present an ethical issue in continuing the study? This may include adverse events, but also study conduct and protocol violations
2. Are there overwhelming data suggesting the superiority of one arm that may present an ethical issue in continuing the study?
  (a) Using the Haybittle-Peto rule, and 90-day all-cause mortality as the outcome of interest, the study will be stopped early if there is a difference in 90-day mortality rate with a *P* value of less than or equal to 0.001
3. Are there any other factors that may impact on the feasibility/usefulness of the study? For example, rate of enrolment, unexpected low rate of outcomes, unable to fund, protocol violations, etc.

### Monitoring and trial co-ordination

#### Trial co-ordination

This trial will be co-ordinated from the Menzies School of Health Research in Darwin (CIs Davis, Tong and Chatfield, and study co-ordinator #1), in collaboration with the Singapore Infectious Diseases Clinical Research Network (CI Lye and study co-ordinator #2). The study will also have input from the Australasian Society for Infectious Diseases (ASID) Clinical Research Network (CRN) and the Australian Kidney Trials Network (AKTN).

#### Data Safety and Monitoring Board

An independent DSMB will be established to review the progress of the study and monitor adherence to the protocol, participant recruitment, outcomes, complications, and other issues related to participant safety. They will also monitor the assumptions underlying sample size calculations for the study and alert the investigators if they see substantial departures as the data accumulate. The DSMB will be composed of experts in infectious diseases, biostatistics and clinical trials. The DSMB members will all be independent of the investigators (none of them will be CIs or site investigators).

The DSMB will make recommendations as to whether the study should continue or be terminated, consider participant safety or other circumstances as grounds for early termination, including either compelling internal or external evidence of treatment differences or feasibility of addressing the study hypotheses (e.g. poor participant enrolment, poor adherence).

#### Study monitoring

Study monitoring will be provided by the responsible monitor(s) at the Menzies School of Health Research (or designee) in accordance with the Monitoring Plan and International Conference on Harmonisation of Technical Requirements for Registration of Pharmaceuticals for Human Use: Good Clinical Practice.

The responsible monitor will visit each study site at least once per year and will be allowed, on request, to inspect the various records (source documents, paper CRFs, electronic case report forms (eCRFs) and other pertinent data) provided that subject confidentiality is maintained in accord with local requirements.

It will be the monitor’s responsibility to inspect the eCRFs throughout the study, to verify the adherence to the protocol and the completeness, consistency and accuracy of the data being entered on them. The monitor must verify that the subject received the study drug as randomised. The monitor should have access to laboratory test reports and other subject records needed to verify the entries on the eCRF. The site PIs agree to co-operate with the monitor to ensure that any problems detected in the course of these monitoring visits are resolved in a timely manner.

### Safety

All trial medications are licensed for use in Australia, Singapore, New Zealand, and Israel with established safety profiles.

#### Serious adverse events (SAEs)

A SAE is defined as any experience that:

- Results in death
- Is life-threatening
  - ○ The term ‘life-threatening’ refers to an event in which the subject was at risk of death at the time of the event. It does not refer to an event, which hypothetically may have caused death, if it were more serious
- Results in unexpected prolongation of existing hospitalisation
- Results in persistent or significant disability/incapacity
- Is a medically important event or reaction

In this trial, expedited reporting of SAEs to HREC will only be required if they are thought by the reporting clinician (the site PI or their delegate) to be related to the intervention arm study drugs (possibly, probably or definitely as defined above). Such SAEs will be reported on the SAE Reporting Form by the site PI or delegate to the sponsor or delegate within 24 hours of the site study team becoming aware of it. The site PI will also report the SAE to the lead HREC for their site within 72 hours. If it is also an unexpected drug reaction, the sponsor or delegate will report to the Therapeutic Goods Administration (TGA).

#### Adverse drug reactions (ADRs)

Investigators will be asked to report all suspected ADRs (regardless of severity or seriousness) which are thought to be related to study drugs in both intervention and control arms (including vancomycin, daptmoycin, flucloxacillin, cloxacillin and cefazolin). These data will be collected routinely on CRF5.

#### SUSARS (suspected unexpected serious adverse drug reactions)

ADRs which are serious (as defined for SAEs above) *and* are unexpected (as defined by not being listed as an adverse effect in the approved product information) *and* are related to the intervention arm study drug (i.e. the β-lactam) will qualify for expedited reporting to the sponsor. As for SAEs, the site PI or their delegate will also report the SUSAR to the HREC within 72 hours. In addition, the sponsor will report the SUSAR to the TGA within 7 calendar days for fatal and life-threatening unexpected serious ADRs, and within 15 calendar days for other serious ADRs.

#### Causality

The site PI will make a judgement regarding whether an adverse event is clinically significant and whether or not it is related to the allocated treatment. The degree of certainty with which an adverse event is attributable to treatment or an alternative cause will be determined by how well the event can be understood in terms of:

- The temporal relationship with the administration of the treatment or cessation of treatment
- Reactions of a similar nature previously observed in the individual or others following treatment

The relationship of the adverse event to treatment will be specified as follows:

Not related:  In the PI’s opinion, there is not a causal relationship
Unlikely:  The temporal association between treatment and the adverse event is such that treatment is not likely to have any reasonable association
Possibly:  The adverse event could have been caused by treatment
Probably:  The adverse event follows a temporal sequence from the time of treatment and cannot be reasonably explained by the known characteristics of the subject’s clinical presentation/history
Definitely:  The adverse event follows a reasonable temporal sequence from the time of treatment or reappears when the treatment is repeated

#### Non-expedited reporting of adverse events and adverse drug reactions

In addition to the expedited reporting described above, a summary of all ADRs (to any of the study drugs including vancomycin, daptomycin or the beta-lactams), including SAEs and SUSARs, will be provided to the HREC and DSMB on a regular basis for review, with the frequency determined by each HREC’s policy.

### Ethical considerations

#### General ethical considerations

The study will be conducted according to the declaration of Helsinki, the Australian National Health and Medical Research Council (NHMRC) criteria for the ethical conduct of research in humans and the principles of GCP [64].

All antimicrobials in this study are registered for use in Australia, Singapore, New Zealand, and Israel. The intervention (the addition of β-lactam to standard therapy) is unlikely to cause harm, and has proven safe both in published human studies and in our own pilot RCT. Furthermore, this combination is routinely used in participants with SAB prior to the availability of antibiotic susceptibility results. MRSA-B is a common condition whose outcomes remain unacceptable with current therapies and this fact along with the strong in-vitro and in-vivo signals justify the conduct of this RCT. Written informed consent will be sought from all participants; in some jurisdictions, consent will be sought from a surrogate decision-maker if the patient is not competent to consent. Approval will be sought from relevant HRECs for all sites. See Appendix for details of approving ethics committees.

### Informed consent

An informed consent discussions will be held with each participant or, for those not competent to make their own decisions (e.g. unconscious), their person responsible. ‘Person responsible’ consent will only be used in jurisdictions where it is allowed, and where the site has research governance approval to do so. The consent process will be carried out by a site investigator or their suitably trained delegate. The information for the discussion will be provided in written and oral formats that have been approved by the HREC and in a language comprehensible to the potential participant or their person responsible, using interpreters if necessary. In the event that a participant who was not competent when initially recruited into the study becomes competent to make their own decisions, the participant will have the study explained to them and an opportunity to consent to remaining in the study or to withdraw. The site investigator or delegate will regularly check to see if the participant becomes competent.

The participant or person responsible will personally sign and date the latest approved version of the consent form, as will the site investigator or their delegate who conducted the consent discussion. If one was used, an interpreter will also sign and date the consent form. A copy of the information statement and consent form will be provided to the participant. No trial-related procedure will be undertaken before documented informed consent is obtained. An original copy of the consent form will be retained at the recruitment site by the site investigator.

### Dissemination policy

The trial results will be communicated to all site PIs prior to publication or presentation. The trial results will be presented at national and international scientific conferences. The trial results will also be submitted for publication to a peer-reviewed scientific journal, irrespective of the results. A plain-language summary of the trial results will be made available to individual participants upon request. The decision where to publish will be made by the Study Steering Committee. The authorship of the paper will include all of the Study Steering Committee who meet International Committee of Medical Journal Editors (ICJME) criteria for authorship. Contributions of other study participants will be recognised by the following language at the end of the named authors’ list: ‘. . . and the CAMERA2 study group for the ASID Clinical Research Network’. The CAMERA2 study group will consist of all named site investigators, and will be listed in the collaborators section of the paper.

## Discussion

MRSA-B is common (over 2880 cases each year in Australia, and 80,000 in the USA), and has an unacceptably high mortality (20–30 %) despite modern supportive care. Indeed, deaths from invasive MRSA infections now outnumber deaths from HIV in the United States [65]. Mortality rates are even higher (50 %) in resource-limited settings [5]. For the past 20 years, researchers and clinicians have been searching for an alternative treatment to vancomycin for MRSA-B [14]. Several new and expensive drugs have been marketed for this indication, but none has proved superior to vancomycin. β-Lactam combination antibiotic therapy has the potential to improve outcomes from MRSA-B, at a very small cost.

There is increasing interest internationally in the role of β-lactam combination therapy for invasive MRSA infections, but most research has thus far focussed primarily on in-vitro laboratory work. This current study will be the first RCT with key clinical endpoints to address this important question, and hence the result (whether it be positive or negative) will influence practice around the world. We have designed the trial with generalisability in mind; the results will apply to patients on haemodialysis (in whom *S. aureus* infections are an important problem, but are often excluded from such trials), patients in wealthy regions including the USA and Europe where daptomycin is commonly used, and, potentially, patients in resource-limited regions, who would be able to afford vancomycin plus flucloxacillin.

### Trial status

The CAMERA2 trial opened its first site for recruitment on 24 August 2015 and randomised its first patient on 26 August 2015.

## Appendix

Appendix for CAMERA2 – combination antibiotic therapy for methicillin-resistant *Staphylococcus aureus* infection: study protocol for a randomised controlled trial.

Names of ethical review committees which have reviewed and approved the CAMERA2 study:

1. Hunter New England Human Research Ethics Committee. This covers all sites in New South Wales, Queensland, Victoria and South Australia. Reference number 15/02/18/3.05
2. Human Research Ethics Committee of the Menzies School of Health Research and Northern Territory Department of Health. This covers the Royal Darwin Hospital site. Reference number HREC 2015-2339
3. Royal Perth Hospital Ethics Committee. Covers all WA sites. Reference number REG 15-008
4. New Zealand Central Health and Disability Ethics Committee. Covers all New Zealand sites. Reference number 15/CEN/95
5. Singapore National Health Group Domain Specific Review Board – Domain E. Covers Singapore sites. Reference Number 2015/00508
6. Human Research Ethics Committee of Rambam Health Corporation. Covers Rambam Hospital Israel. Reference Number 0322-15-RMB

## Abbreviations

ADR: adverse drug reaction
AE: adverse event
AKTN: Australian Kidney Trials Network
ANZCOSS: Australian and New Zealand Co-operative Outcomes of Staphylococcal Sepsis
ASID CRN: The Australian Society for Infectious Diseases Clinical Research Network
β-lactams: a family of related antibiotics which includes the penicillins cephalosporins and carbepenems
CI: chief investigator – a researcher who contributes to the funding planning, and running of the entire study
CK: creatinine kinase
CNS: coagulase-negative *Staphylococcus*
Co-I: co-investigator (a clinician or research assistant who aids the PI at a site)
CRP: C-reactive protein
DNA: deoxyribonucleic acid
DSMB: Data Safety Monitoring Board
EDC: Electronic Data Capture
EUC: electrolytes urea and creatinine
eCRF: electronic case report forms
FBC: full blood count
GCP: Good Clinical Practice
GFR: glomerular filtration rate
GP: general practitioner
HRN: hospital record number
HREC: Human Research Ethics Committee
hVISA: heterogeneous vancomycin-intermediate resistance *Staphylococcus aureus*
ICH: International Conference on Harmonisation
ICU: intensive care unit
ID: identification
ID physician: infectious disease physician
IDSA: Infectious Disease Society of America
Index blood culture: the first positive MRSA blood culture taken from the patient for that clinical episode
IRB: Institutional Review Board
IV: intravenously
IVI: intravenous infusion
LFT: liver function test
MIC: minimum inhibitory concentration
MRSA: methicillin-resistant *Staphylococcus aureus*
MRSA-B: MRSA bacteraemia
MSSA: methicillin-sensitive *Staphylococcus aureus*
MSSA-B: MSSA bacteraemia
NHMRC: National Health and Medical Research Council
NOK: next of kin
PI: principal investigator (a clinician responsible for one site)
QID: four times per day
RCT: randomised control trial
RIFLE criteria: acronym for Risk Injury, and Failure and Loss and End-stage kidney disease (classification system for acute kidney injury)
SAB: *Staphylococcus aureus* bacteraemia
SAE: serious adverse events
SCRN: The Singapore Infectious Diseases Clinical Research Network
SUSAR: suspected unexpected serious adverse reaction
TDS: three times per day
TGA: Therapeutic Goods Administration
VISA: vancomycin intermediate *Staphylococcus aureus*
VRSA: vancomycin-resistant *Staphylococcus aureus*
